# A10 OVEREXPRESSION OF ASCL2 ALTERS DIFFERENTIATION, CELL CYCLE AND RESISTANCE TO ANTI-CANCER TREATMENT IN ESOPHAGEAL ORGANOIDS

**DOI:** 10.1093/jcag/gwac036.010

**Published:** 2023-03-07

**Authors:** M Hamilton, Z Mars, M Sedeuil, M Rolland, D Jean, V Giroux

**Affiliations:** Immunologie et de Biologie cellulaire, Université de Sherbrooke, Sherbrooke, Canada

## Abstract

**Background:**

The esophagus is in constant contact with the austere environment caused by food and gastric reflux. It is protected by a squamous epithelium which maintenance is provided by a rare subpopulation of basal cells: *Keratin 15+* (*Krt15+*) stem cells. However, little is known about the mechanisms underlying the expansion and the function of these stem cells. It was shown that the transcription factor ASCL2 is strongly upregulated in *Krt15*+ cells compared to *Krt15*- cells. Interestingly, ASCL2 is a gene target of the Wnt/β-catenin pathway, which acts as a regulator of proliferation and maintenance of the stemness state.

**Purpose:**

The ultimate goal of my research project is to determine the role of ASCL2 in the maintenance of esophageal stem cells. To do so, I will investigate the role of ASCL2 in esophageal epithelial biology.

**Method:**

Lentiviral infection approach was used to obtain mouse esophageal organoids overexpressing ASCL2 (ASCL2^OE^). Organoid culture, immunostaining (such as IF and H&E), qPCR, WB, mass spectrometry and proliferation assay were used to characterize the effect of ASCL2^OE^ on morphology, differentiation, proliferation, self-renewal, and gene expression.

**Result(s):**

ASCL2^OE^ severely altered the morphology of organoids, which were smaller and less differentiated. Defects in differentiation was investigated by IF which showed that some cells expressed both p63 and K13, respectively basal and suprabasal markers. Thus, cells seem to be blocked in an intermediate state of differentiation suggesting a default in cell fate decision. Mass spectrometry analysis confirmed a change in biological processes related to differentiation of keratinocytes and of epithelial cells. We also investigated the role of ASCL2 in self-renewal and observed that organoid formation rate (OFR) was reduced in ASCL2^OE^ organoids. Furthermore, proliferation was also reduced in WST-1 and EdU assays. We then observed significant changes in the cell cycle by flow cytometry: there is an increased in the number of cells in G0/G1 and a major decrease in G2/M cells, suggesting a blockade in G1. Interestingly, CDNK2a (p16INK4a), an inhibitor of cell cycle progression, was increased in our mass spectrometry results. Finally, ASCL2 could also play a role in radio and chemoresistance of *Krt15*+ stem cells, as ASCL2^OE^ organoids are less sensitive to radiation and chemotherapy agents than control.

**Conclusion(s):**

ASCL2 could play a role in orchestrating cell fate decision in the esophageal epithelium as ASCL2^OE^ organoids showed alteration in differentiation, proliferation, and cell cycle.

**Please acknowledge all funding agencies by checking the applicable boxes below:**

Other

**Please indicate your source of funding;:**

NSERC, Canada Research Chairs and CRCHUS scolarship

**Disclosure of Interest:**

None Declared

